# Neoadjuvant Chemotherapy with Laser Interstitial Thermal Therapy in Central Nervous System Neuroblastoma: Illustrative Case and Literature Review

**DOI:** 10.3390/brainsci13111515

**Published:** 2023-10-26

**Authors:** Jason E. Chung, Omar Iqbal, Chandra Krishnan, Virginia Harrod, Elizabeth Tyler-Kabara, Rongze O. Lu, Winson S. Ho

**Affiliations:** 1 Department of Neurological Surgery, University of California, San Francisco, CA 94110, USA; jason.chung@ucsf.edu (J.E.C.); rongze.lu@ucsf.edu (R.O.L.); 2 Department of Neurosurgery, Dell Medical School, University of Texas at Austin, Austin, TX 78712, USA; omar.iqbal@austin.utexas.edu (O.I.); elizabeth.tylerkabara@austin.utexas.edu (E.T.-K.); 3 Department of Diagnostic Medicine, Dell Medical School, University of Texas at Austin, Austin, TX 78712, USA; ckrishnan@ascension.org; 4 Department of Pediatrics, Dell Medical School, University of Texas at Austin, Austin, TX 78723, USA; vlharrod@ascension.org

**Keywords:** CNS neuroblastoma, laser interstitial thermal therapy, LITT

## Abstract

Primitive neuroectodermal tumors of the central nervous system, or CNS neuroblastoma, are rare neoplasms in children. Recently, methylation profiling enabled the discovery of four distinct entities of these tumors. The current treatment paradigm involves surgical resection followed by chemotherapy and radiation. However, upfront surgical resection carries high surgical morbidity in this patient population due to their young age, tumor vascularity, and often deep location in the brain. We report a case of CNS neuroblastoma that can be successfully treated with neoadjuvant chemotherapy followed by minimally invasive laser interstitial thermal therapy and radiation. The patient has complete treatment with no evidence of recurrence at one year follow-up. This case illustrates a potential paradigm shift in the treatment of these rare tumors can be treated using minimally invasive surgical approach to achieve a favorable outcome.

## 1. Introduction

Primitive neuroectodermal tumors of the CNS (CNS-PNETs) are neoplasms that primarily affect children. These tumors are characterized by small, poorly differentiated embryonal cells making histologic diagnosis challenging due to the lack of defining molecular markers. Recently, methylation profiling enabled the identification of four distinct new CNS-PNET entities, each associated with a recurrent genetic alteration and distinct clinical features [1], one of the entities is CNS neuroblastoma with forkhead box R2 (FOXR2) activation (CNS NB-FOXR2). Limited clinical data suggest that CNS NB-FOXR2 has a more favorable outcome than other CNS-PNETs. Current treatment involves surgical resection followed by combination of chemotherapy and radiation [2,3,4,5,6]. However, these tumors are often large and vascularized. In the pediatric population with low blood volume, the surgical morbidity of attempting gross total resection is significant. Laser interstitial thermal therapy (LITT) is a minimally invasive approach to achieve an ablative lesion in the brain that has seen increasing adoption for treatment of epilepsy and brain tumors with a favorable complication profile compared to traditional open resection [7,8]. LITT destroys the target tissue through the use of thermal energy from a stereotactically inserted laser probe. Multiple fibers can be inserted to achieve the desired ablative volume. In this case, we highlight the value of a precision oncology approach that identified this favorable entity and proceeded with neoadjuvant chemotherapy followed by LITT of the residual tumor, which achieved a good outcome with minimal surgical morbidity.

## 2. Methods

Participant—The patient was treated with 4 cycles of neoadjuvant chemotherapy as per the Children’s Oncology Group Protocol ACNS0334, Induction Regimen A (Vincristine, Cyclophosphamide, Etoposide, Cisplatin), followed by LITT for surgical cytoreduction. She then resumed therapy with craniospinal radiation IMRT with concurrent vincristine. Treatment then continued with consolidation therapy with 2 cycles of ACNS0332 (vincristine, cyclophosphamide, cisplatin).

Materials—Tumor samples were obtained via surgery performed by a neurosurgeon (W.H.). FFPE specimens derived from fresh tumor biopsies were reviewed by a pathologist (C.K.). Tumor samples were evaluated using hematoxylin and eosin staining and immunohistochemical staining. DNA was extracted, purified, and quantified. For genomic analysis, the sample was sent to Mayo Laboratories for the MayoComplete Next Generation Sequencing (NGS) neuro-oncology panel for the detection of mutations in the coding sequence and rearrangements in 160 genes (Mayo Medical Center, Rochester, MN, USA). For methylation profiling, the sample was sent to NIH NINDS/NOB laboratory using the Illumina Infinium Human Methylation 450 Bead-Chip and analyzed using the Heidelberg (DKFZ)-developed validated DNA methylation classifier. All procedures were performed in a CLIA-compliant environment.

## 3. Results

### Case Report

**Figure 1:**
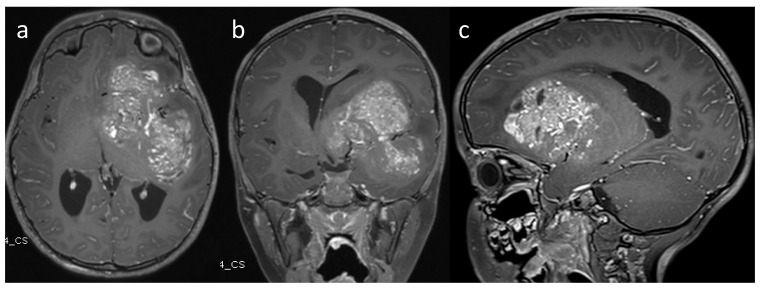
(**a**) Axial, (**b**) coronal, and (**c**) sagittal contrast-enhanced MRI at the time of initial diagnosis demonstrated a large multi-lobular, contrast-enhancing, mass-causing mass effect; midline shift; and obstructive hydrocephalus.

**Figure 2:**
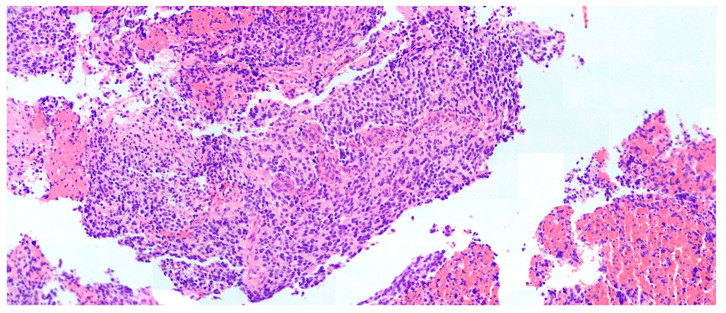
Hematoxylin and eosin stain of tumor specimen demonstrated an infiltrative hypercellular embryonal neoplasm without specific architectural patterns.

**Figure 3:**
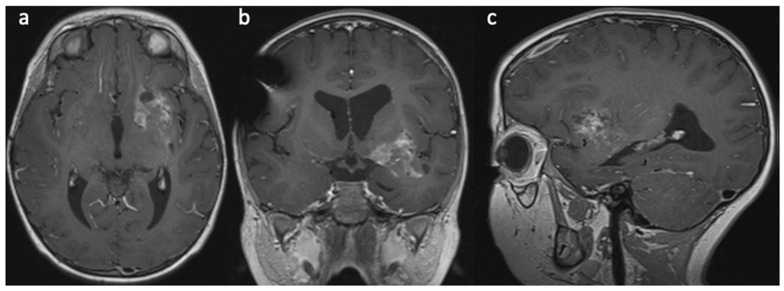
(**a**) Axial, (**b**) coronal, and (**c**) sagittal contrast-enhanced MRI after 4 cycles of neoadjuvant chemotherapy as per the Children’s Oncology Group Protocol ACNS0334, Induction Regimen A (Vincristine, Cyclophosphamide, Etoposide, Cisplatin), demonstrated significant reduction in the size of the contrast enhancing lesion.

**Figure 4:**
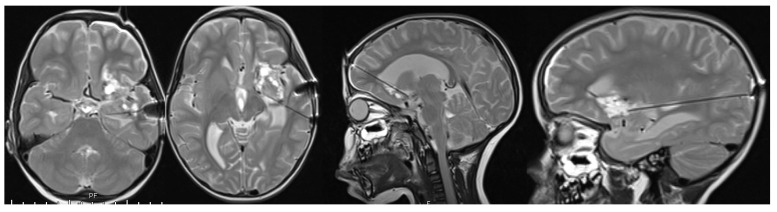
Intraoperative MRI confirming placement for 4 Medtronic Visualase fiber prior to ablation of tumor. Two fibers were inserted into the temporal portion of the tumor laterally. One fiber inserted into the posterior portion of the tumor from an occipital approach. One fiber inserted into the anterior portion of the from a frontal approach.

**Figure 5:**
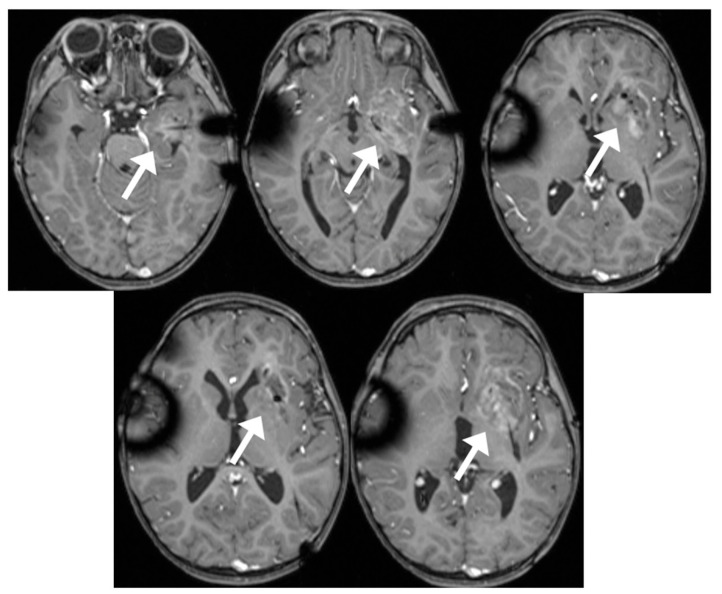
Serial axial contrast-enhanced MRI after LITT demonstrating area of post-treatment necrosis (arrows), reflecting the ablated area.

**Figure 6:**
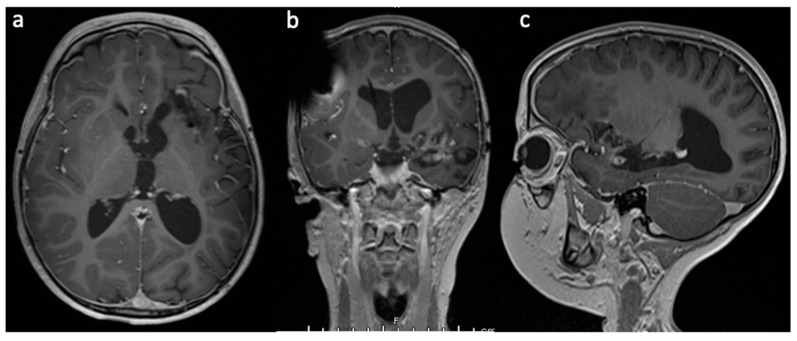
(**a**) Axial, (**b**) coronal, and (**c**) sagittal contrast-enhanced MRI after completion of LITT, post LITT craniospinal radiation IMRT, and consolidation chemotherapy 13 months after initial diagnosis. There is no evidence of tumor recurrence.

A 4-year-old female with no significant past medical history. She presented with 6 weeks of progressive right-sided body weakness and nausea/vomiting worse in the morning. The physical exam was notable for papilledema, right facial droop, and right-sided body weakness 4-/5. A brain MRI demonstrated a large, aggressive-appearing heterogeneously enhancing mass centered in the left frontotemporal lobes with solid and cystic features as well as evidence of prior hemorrhage causing complete effacement of the 3rd ventricle and trapping of the lateral ventricles with 1 cm of midline shift (Figure 1). Due to the size, involvement of deep structures, and evidence of marked vascularity, a decision was made to proceed with a stereotactic needle biopsy and placement of a ventriculoperitoneal shunt to address the hydrocephalus. The biopsy tissue demonstrated an infiltrative hypercellular embryonal neoplasm without specific architectural patterns (Figure 2). The tumor cells exhibited positive staining for synaptophysin and S100 along with weak membranous reactivity with Alk-1. Next-generation sequencing (NGS) performed by Mayo Medical Laboratories (Mayo Medical Center, Rochester, MN, USA) did not reveal any pathognomonic mutations or alterations seen in genes associated with pediatric brain tumors. Given the diagnostic uncertainty, tumor tissue was sent to the NIH for methylation profiling, which demonstrated a high-confidence profiling match with CNS neuroblastoma-FOXR2 activation using version 11 of the CNS classifier. Given the concern for surgical morbidity to attempt a resection of this large mass in a 4-year-old and the reported sensitivity to chemotherapy in this subgroup of CNS-PNETs, we considered neoadjuvant chemotherapy as described in a small series of rare infantile brain tumors [9,10] with the hope that chemotherapy would de-vascularize and shrink the tumor to render surgery safer. The patient was treated with neoadjuvant chemotherapy as per the Children’s Oncology Group Protocol ACNS0334, Induction Regimen A (Vincristine, Cyclophosphamide, Etoposide, Cisplatin). MRI after the fourth cycle of chemotherapy demonstrated a remarkable response in size and vascularity of the mass (Figure 3). However, the residual tumor was centered in the temporal stem with extension towards both the frontal and temporal lobes. An optimal surgical approach would require a transsylvian resection that would put deep structures, such as anterior perforated substance, and deep vessels, such as the choridal artery that supplies the internal capsule, at risk. Given that the available data suggest that CNS NB-FOXR2 carries a more favorable outcome and that the benefits of cytoreduction/extent of resection have not been proven in these entities, we elected to attempt LITT of the residual mass. Four laser fibers were placed using robotic stereotactic assistance (ROSA) robotic guidance to provide adequate coverage to ablate the entirety of the residual lesion (Figure 4). Ablation was carried out using the Medtronic Visualase system. Post-ablation MRI demonstrated that the residual tumor was completely covered by the ablative zone (Figure 5). Post-operatively, the patient was at her neurologic baseline and was discharged home 3 days after surgery. After surgical intervention, she resumed therapy with craniospinal radiation IMRT 54Gy with 30.6Gy boost to the tumor bed with concurrent vincristine. Treatment then continued with consolidation therapy with 2 cycles of ACNS0332 (vincristine, cyclophosphamide, cisplatin). She was last seen 6 months post-completion of treatment (13 months after initial presentation) and was doing well clinically without evidence of disease recurrence on MRI (Figure 6).

## 4. Discussion

CNS PNETs consist of a diverse set of tumor types. The CNS NB-FOXR2 tumor subtype was first identified in Sturm et al. 2016 [1] using methylation profiling. This subset was integrated into the World Health Organization (WHO) Classification of CNS Tumors in 2021 [11]. Since then, clinical data of this rare tumor subtype is limited to relatively small series, the largest being a cohort of 63 patients reported in von Hoff et al. [4]. Median age at diagnosis for patients with CNS NB-FOXR2 is 5 years old, with the vast majority of cases presenting between age 2 and 6 across all reported studies [4,6,12]. There is a slightly higher number of females compared to males (36 vs. 27). A minority of cases (10/63) had intracranial or spinal metastases [4]. Imaging characteristics [12,13] of these tumors often involve the deep white matter and cortex of one or multiple frontal/parietal/temporal lobes, as well as the basal ganglia (68% involving frontal lobe, 40% parietal, 40% temporal, 15% occipital; 40% basal ganglia, 15% thalamus; 85% involving cortex, 97% involving deep white matter). Masses tended to be large, with non-solid components, multilobulated, intermediately enhancing, and with little surrounding edema [13]. Besides the tumor-defining activation of FOXR2 on methylation studies, the pathology of CNS NB-FOXR2 is characterized by highly cellular tumor mass of small round cells with hyperchromatic nuclei, densely packed tissue with neuroblastic-like rosettes, islands of neuropil, robust microvascular proliferation, and strong olig2 immunoexpression [14].

Treatment for CNS NB-FOXR2 typically follows a trial-based CNS embryonal tumor treatment pathway, which typically begins with surgery followed by combination of chemotherapy and/or radiation. In von Hoff et al. [4], all 63 patients underwent resective surgery; 23/63 received an initial gross total resection, while 27/63 had incomplete resection. Of the 27 who had incomplete resection, 6 had repeat resective surgery and 5/6 had a gross total resection on second-look surgery. Following surgery, 44/63 underwent chemotherapy, and 18/63 underwent craniospinal radiation followed by chemotherapy. It was suggested that CNS NB-FOXR2 tumors are more chemo-sensitive than other tumors previously under the larger “PNET” categorization [4] and carry an improved prognosis (63 patients, 63% 5-year PFS: 63% ± 7%, OS: 85% ± 5%). A recent retrospective case series found a 5-year progression-free survival of 100% (7 patients) [6].

The patient discussed here is a relatively common presentation of this rare tumor. The deep location, hypervascular nature, and involvement of the basal ganglia and temporal stem made upfront surgical resection in this age group with low blood volume highly risky. We therefore first proceeded with a stereotactic biopsy, which demonstrated CNS NB-FOXR2 pathology. Given the reported increased chemo-sensitivity in this genetic subgroup and drawing from our experience and that of others in using neoadjuvant chemotherapy in rare infant brain tumor types, we proceeded with upfront chemotherapy. The tumor had a remarkable response, and the residual volume was amenable to LITT, allowing for a minimally invasive approach to achieving cytoreduction. 

LITT is increasingly being used in treatment for adult and pediatric brain tumors in both newly diagnosed and recurrent settings [15,16,17,18], but no head-to-head comparison is currently available to demonstrate non-inferiority compared to surgical resection. LITT uses thermal destruction of target tissue through the use of heat from inserted laser probes. MRI thermography allows for real-time feedback and monitoring of the thermal ablation. Combined with the placement of the intracranial laser probe using stereotaxy, LITT provides an extremely accurate alternative to open surgery. The Monteris NeuroBlate and Medtronic Visualase are the two FDA-approved LITT platforms in North America. For lesions in areas that are deep and difficult to access surgically, the favorable complication profiles of LITT make it a compelling alternative such as in this patient. This illustrative case is, to our knowledge, the first to describe the use of neoadjuvant chemotherapy followed by LITT for any brain tumors. This combination is particularly appealing for this rare entity CNS NB-FOXR2, which often are large vascular tumors in young children that confers high surgical risks with upfront resection. In addition, chemotherapy is already part of standard of care treatment in these tumors, suggesting that this may be an appealing strategy for tackling these rare tumors that will minimize treatment morbidity. 

## 5. Conclusions

LITT in combination of neoadjuvant chemotherapy can be an effective treatment strategy for rare pediatric CNS neuroblastoma, which carries less surgical morbidity than traditional open surgical resection.

## Data Availability

Samples were sequenced and analyzed in a CLIA-compliant Mayo and NIH laboratory as described above. The raw sequencing data are not publicly available due to data privacy regulations and restrictions for use of such data, as stated in the patient consent form.
